# Small molecule activators of SIRT1 replicate signaling pathways triggered by calorie restriction *in vivo*

**DOI:** 10.1186/1752-0509-3-31

**Published:** 2009-03-10

**Authors:** Jesse J Smith, Renée Deehan Kenney, David J Gagne, Brian P Frushour, William Ladd, Heidi L Galonek, Kristine Israelian, Jeffrey Song, Giedre Razvadauskaite, Amy V Lynch, David P Carney, Robin J Johnson, Siva Lavu, Andre Iffland, Peter J Elliott, Philip D Lambert, Keith O Elliston, Michael R Jirousek, Jill C Milne, Olivier Boss

**Affiliations:** 1 Sirtris, a GSK company 200 Technology Square, Cambridge, MA, 02139, USA; 2 Genstruct Inc, One Alewife Center, Cambridge, MA, 02140, USA

## Abstract

**Background:**

Calorie restriction (CR) produces a number of health benefits and ameliorates diseases of aging such as type 2 diabetes. The components of the pathways downstream of CR may provide intervention points for developing therapeutics for treating diseases of aging. The NAD^+^-dependent protein deacetylase SIRT1 has been implicated as one of the key downstream regulators of CR in yeast, rodents, and humans. Small molecule activators of SIRT1 have been identified that exhibit efficacy in animal models of diseases typically associated with aging including type 2 diabetes. To identify molecular processes induced in the liver of mice treated with two structurally distinct SIRT1 activators, SIRT501 (formulated resveratrol) and SRT1720, for three days, we utilized a systems biology approach and applied Causal Network Modeling (CNM) on gene expression data to elucidate downstream effects of SIRT1 activation.

**Results:**

Here we demonstrate that SIRT1 activators recapitulate many of the molecular events downstream of CR *in vivo*, such as enhancing mitochondrial biogenesis, improving metabolic signaling pathways, and blunting pro-inflammatory pathways in mice fed a high fat, high calorie diet.

**Conclusion:**

CNM of gene expression data from mice treated with SRT501 or SRT1720 in combination with supporting *in vitro *and *in vivo *data demonstrates that SRT501 and SRT1720 produce a signaling profile that mirrors CR, improves glucose and insulin homeostasis, and acts via SIRT1 activation *in vivo*. Taken together these results are encouraging regarding the use of small molecule activators of SIRT1 for therapeutic intervention into type 2 diabetes, a strategy which is currently being investigated in multiple clinical trials.

## Background

Calorie restriction (CR), defined as a reduction of caloric intake with adequate nutrition, ameliorates common diseases of aging, such as insulin resistance, type 2 diabetes mellitus (T2DM), dyslipidemia, neurodegenerative disorders and cancer [1-5]. CR is effective in extending lifespan in most every organism examined to date, including yeast, drosophila, and several mammalian species [4,6-8]. The mechanism underlying these beneficial effects is not well understood and elucidation of the molecular signaling pathways downstream of CR might enable pharmacological intervention into this pathway [9,10].

Silent information regulator 2 (*SIR2*) was first identified as a mediator of CR-induced lifespan extension in yeast [11]and subsequently has been shown to regulate this process in lower metazoans, such as *Drosophila melanogaster *and *C. elegans *[12-14]. The mammalian ortholog of Sir2, SIRT1, is an NAD^+^-dependent protein deacetylase that catalyzes the removal of acetyl groups from lysine residues in substrate proteins. SIRT1 deacetylates a number of cellular proteins including p53, FOXO, PGC-1α, and LXR [15-22]. Following a regimen of CR in both rodents and in humans, SIRT1 expression levels are increased [23-26]. SIRT1 belongs to the sirtuin family that consists of seven enzymes that share a conserved core catalytic domain but differ in their flanking amino- and carboxy-terminal sequences, cellular localization, tissue distribution, and substrate proteins [27-29]. Increasing evidence implicates mammalian sirtuins as regulators of CR-induced physiological responses, including lipid metabolism, glucose homeostasis, stress responses and insulin secretion [29].

As potential mediators of CR *in vivo*, the sirtuin enzymes may represent novel intervention points for developing therapeutics to treat diseases of aging such as type 2 diabetes. In particular, SIRT1 activators have been described and these show promise in improving metabolic profiles in both genetic and diet induced obese rodents. The first SIRT1 activator described, resveratrol, is a polyphenolic compound found in red wine. Resveratrol is a micromolar activator of SIRT1 *in vitro *and has been shown to improve the metabolic profile of mice fed a high fat diet [30,31]. In this model of type 2 diabetes resveratrol increases mitochondrial biogenesis and ameliorates insulin resistance.

Recently, we reported novel small molecule activators of SIRT1 that are 1000 times more potent than resveratrol [32]. These compounds are structurally distinct from resveratrol but act through the same enzymatic mechanism, binding to an allosteric site exposed in the enzyme-substrate complex and thereby lowering the K_m _of SIRT1 for its acetylated peptide substrates. Most notably, these molecules exhibit good oral bioavailability in rodents and like resveratrol, improve both glucose and insulin homeostasis in *ob/ob *mice, diet induced obese mice, and Zucker *fa/fa *rats.

In this report we characterize the early molecular signaling events that are shared between treatment with two structurally distinct SIRT1 activating compounds, SRT501, a proprietary formulation of resveratrol with enhanced pharmacokinetic properties and improved oral bioavailability, and SRT1720, a chemically distinct compound with nanomolar potency toward SIRT1 [32]. Using a Causal Network Modeling (CNM) approach empowered by transcriptional profiling data from mice treated with the SIRT1 activators and a global knowledgebase, we determined that SRT501 and SRT1720 recapitulate a molecular signature which overlaps with that of CR. Furthermore, we characterize several key hypotheses underlying the CR network for SIRT1 activator mechanism of action, and show that metabolism mediated by Ppar family members is upregulated, mitochondrial biogenesis is enhanced, and pro-inflammatory signaling is decreased. In addition, both SRT501 and SRT1720 show significant overlap in the signaling pathways they modulate supporting the concept that these structurally distinct compounds act on the same molecular target, SIRT1, *in vivo*. Finally, we provide complementary *in vivo *and *in vitro *evidence that supports the hypothesis that SIRT1 activation *in vivo *by small molecules mimics calorie restriction.

## Results

**Figure 1:**
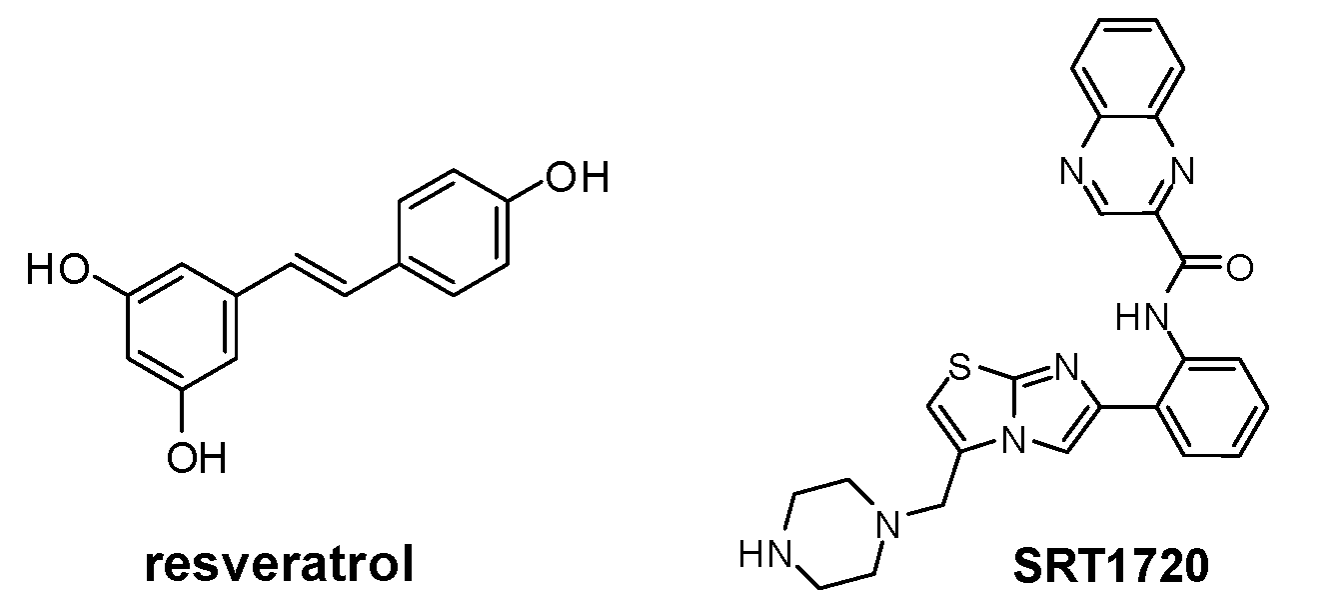
**Chemical Structures of SRT501-resveratrol and SRT1720**.

Small molecule activators of SIRT1 improve metabolic parameters in several rodent models of T2DM. To elucidate the pathways downstream of SIRT1 activation responsible for the observed efficacy in these *in vivo *models, we performed Causal Network Modeling (CNM) of gene expression data from livers of mice fed a high fat diet (diet-induced obesity, DIO) and treated with SIRT1 activators, SRT501 (1000 mg/kg, oral dosing) or SRT1720 (100 mg/kg, oral dosing) for three days (Figure 1). Both compounds exhibit good oral bioavailability in mice.

CNM utilizes a species-specific knowledge assembly model derived from a global knowledgebase (see Additional file 1) as the substrate for automated reverse causal analysis powered by gene expression changes (state changes) from the experiment under analysis (see Materials and Methods for more information)[33,34]. Automated reverse causal analysis generates a list of statistically significant upstream explanations, or hypotheses, for changes in RNA expression in response to SIRT1 activator treatment. These hypotheses are subsequently evaluated for biological integrity and experimental relevance and assembled into causal biological networks. Automated reverse causal analysis identified 146 statistically significant hypotheses that could explain the changes in gene expression observed in the livers of DIO mice treated with SRT501 for 3 days versus controls (SRT501 dataset comparison). Three key pathways, comprised of 12 of the 146 hypotheses are the focus of this report: 1) calorie restriction-resveratrol treatment, 2) increased metabolism and mitochondrial biogenesis and 3) decreased pro-inflammatory signaling. These pathways were chosen because to date, they have been validated by *in vitro *and *in vivo *experimental data. Initially, we characterized the molecular pathways induced by SRT501 and in order to determine if the mechanisms identified for SRT501 treatment were congruous with SRT1720 treatment, the directionality of the individual expression changes supporting the hypotheses identified from SRT501 treatment were compared to those observed after SRT1720 treatment using a statistical comparison at the probe set level. Finally, the results of our modeling efforts were confirmed by *in vitro *and *in vivo *experimental data.

Statistical analysis of the microarray data identified 342 genes whose RNA expression was significantly modulated in response to SRT501 treatment. While all molecular state changes can be mapped to the knowledgebase, only the subset of these changes that have one or more upstream causal assertions in the knowledgebase are considered causally modeled. If there is no information in the knowledgebase for the transcriptional control of a given gene, then it is not possible to identify hypotheses that can explain this state change. In the case of the SRT501 dataset, 78% (266 of 342) of the state changes were causally modeled. The high percentage of modeled state changes indicates that the knowledgebase is sufficiently enriched with causal information about the experimental outcome to be competent for reverse causal analysis.

### SRT501 and SRT1720 generate a gene expression profile similar to that of calorie restriction

**Figure 2:**
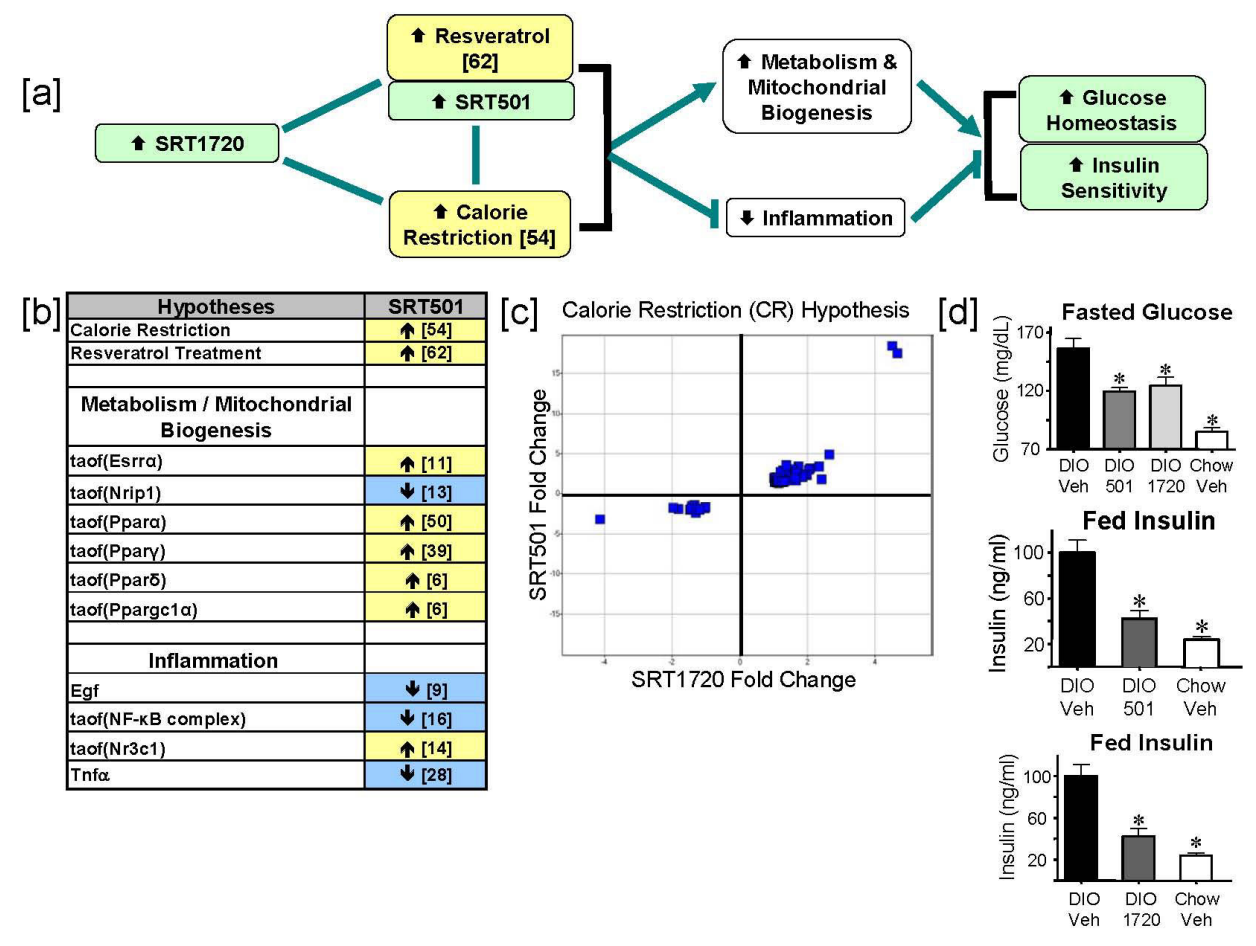
**SRT1720 behaves as a mimetic for calorie restriction and improves glucose homeostasis and insulin sensitivity**. [a] Flow diagram depicting the downstream effects of Sirt1 activation by the compounds SRT501 and SRT1720 in DIO mouse livers after 3 days of treatment. Green – measured and observed increase. Yellow – statistically significant hypothesized increase. White – a given process (e.g. inflammation) supported by statistically significant hypotheses. Blue – hypothesized decrease in activity or abundance. Non-directional lines indicate similarity between SRT501, SRT1720 and resveratrol treatment and the effects of calorie restriction. Lines with arrowheads indicate causal activation; lines with bars indicate causal inhibition. The direction of the arrow in boxes indicates whether a process, hypothesis or observation shows an increase or decrease with SRT501 or SRT1720. Numbers in brackets indicate the number of RNA state changes supporting that hypothesis. [b] Table of processes activated or attenuated in response to SRT501 and SRT1720 treatment and the statistically significant hypotheses that support them. [c] Scatter plot obtained by graphing fold changes of significant probe sets supporting the increased CR hypothesis in the SRT1720 dataset comparison versus the SRT501 dataset comparison. [d] Fasted plasma glucose levels were improved following treatment with either SRT501 (1000 mg/kg) or SRT1720 (100 mg/kg) as compared to vehicle treated DIO mice (* p < 0.05). Fed plasma insulin following treatment with SRT501 (1000 mg/kg) or SRT1720 (100 mg/kg) was significantly reduced (*p < 0.05) as compared to vehicle treated control DIO mice. Error bars represent standard error of the mean.

One of the strongest hypotheses generated from reverse causal analysis on the SRT501 dataset comparison is CR, supported by 54 independent changes in gene expression, corresponding to ~20% of the causally modeled state changes (Figure 2[a] and 2[b]). Reverse causal analysis on the SRT501 dataset comparison identified two processes downstream from SRT501 treatment that are supported by causal networks of statistically significant hypotheses: the increased metabolism and mitochondrial biogenesis network and the decreased inflammation network (Figure 2[a]). Together, these two networks along with the CR and resveratrol treatment hypotheses (discussed below) make up the larger CR network for SIRT1 activator mechanism of action. To determine whether the CR hypothesis was also supported in the SRT1720 dataset comparison, we analyzed the 73 probe sets mapping to the 54 state changes underlying the CR hypothesis. Importantly, all 73 probe sets were modulated in the same direction in the SRT1720 and SRT501 dataset comparisons (p < 10^-5^, Figure 2[c]) suggesting that these two structurally distinct SIRT1 activators act via the same mechanism *in vivo*. These findings support the hypothesis that SRT1720, like SRT501, induces a gene expression profile similar to CR.

Notably, resveratrol treatment was identified as a statistically significant hypothesis supported by 62 state changes (~23% of the causally modeled state changes). The resveratrol treatment hypothesis is supported by data encoded into the knowledgebase from prior studies demonstrating that this compound recapitulates many of the metabolic benefits of CR in rodent models of obesity and insulin resistance [30,31]. As with the CR hypothesis, analysis of the state changes underlying the resveratrol treatment hypothesis determined that probe sets mapping to these state changes were modulated in the same direction after SRT1720 treatment compared to SRT501 treatment (p < 10^-5^, see Additional file 2).

*In vivo *efficacy data for SRT501 and SRT1720 in multiple rodent models for T2DM also agree with the CR network shown in Figure 2[a], where these compounds exhibit similar effects to calorie restriction. We recently reported that these SIRT1 activators, like calorie restriction, improve glucose and insulin homeostasis in three models of T2DM: a C57BL/6 mouse diet-induced-obesity model, an *ob/ob *mouse model and a Zucker *fa/fa *model [32]. Figure 2[d] demonstrates efficacy of these two compounds in the diet-induced obesity model using C57BL/6 mice. After 3 weeks of dosing mice with SRT501 (1000 mg/kg) and SRT1720 (100 mg/kg), fasted blood glucose is decreased by 24% and 20% in SRT501 and SRT1720 treatment groups respectively compared to vehicle control animals. Similarly, fed insulin levels are decreased by ~60% and ~50% respectively in animals dosed with SRT501 (1000 mg/kg) or SRT1720 (100 mg/kg) compared to vehicle control animals. These data are consistent with our previously reported findings that SRT501 and SRT1720 improve glucose and insulin homeostasis in rodent models of T2DM [32].

### SRT501 and SRT1720 increase metabolic signaling and mitochondrial biogenesis

**Figure 3:**
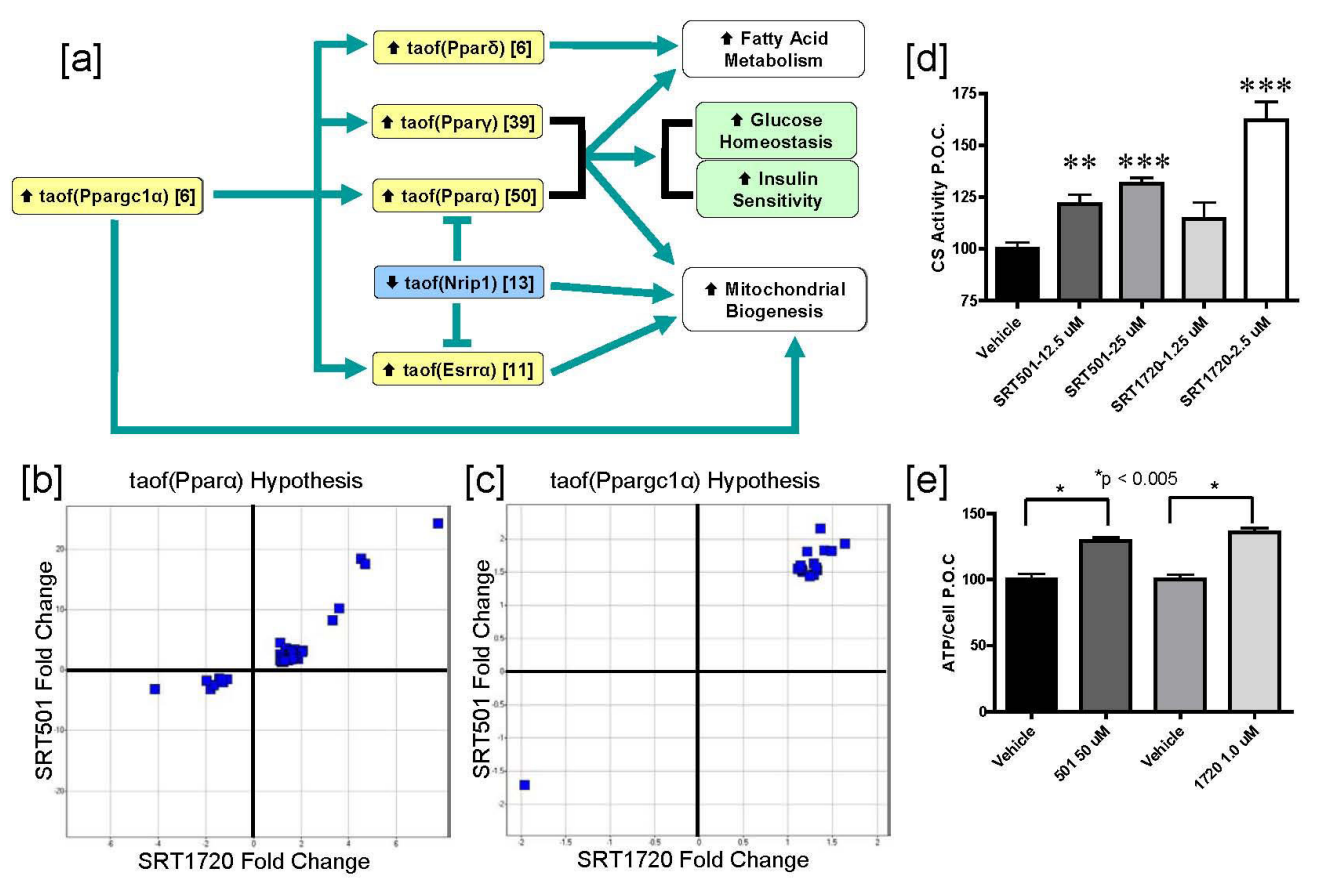
**SRT501 and SRT1720 treatment leads to increased metabolism and mitochondrial biogenesis**. [a] Flow diagram of a network depicting statistically significant hypotheses supporting increased metabolism and mitochondrial biogenesis in DIO mouse livers after 3 days of treatment. Numbers in brackets indicate the number of RNA state changes supporting that hypothesis. taof – *t*ranscriptional *a*ctivity *of *a given protein. [b] Scatter plot obtained by graphing fold changes of significant probe sets supporting the increased taof(Pparα) hypothesis in the SRT1720 dataset comparison versus the SRT501 dataset comparison. [c] Scatter plot obtained by graphing fold changes of significant probe sets supporting the increased taof(Ppargc1α) hypothesis in the SRT1720 dataset comparison versus the SRT501 dataset comparison. [d] C2C12 myotubes were treated with vehicle, SRT501 or SRT1720 at the indicated concentrations for 48 hours. Citrate Synthase activity was measured in lysates as a marker for mitochondrial function. p values were measured using an unpaired, 2-tailed t test. n = 3 replicates per group for compound treatments and n = 6 replicates for vehicle treatment. Error bars represent standard error of the mean. (**p < 0.01, ***p < 0.001). [e] NCI-H358 cells were treated with vehicle, SRT501 (50 μM) or SRT1720 (1 μM) for 48 hours. Cellular ATP levels were then measured as marker for mitochondrial function. P values were measured using an unpaired, 2-tailed t test. n = 3 replicates per group. Error bars represent standard error of the mean (*p < 0.005).

The hypotheses supporting the increased metabolism and mitochondrial biogenesis network downstream of SRT501 treatment are displayed in Figure 3[a]. One key hypothesis supporting this process is increased transcriptional activity of Ppargc1α (Pgc-1α) which is supported by 6 state changes. Several reports have demonstrated that deacetylation of Ppargc1α by SIRT1 at multiple lysine residues leads to increased transcriptional activity of Ppargc1α towards its target genes (Rodgers et al 2005). Furthermore, Ppargc1α is known to function as a coactivator for nuclear hormone receptors, such as Ppar α,γ,δ and Esrrα (Errα), all of which are predicted to have increased transcriptional activities in the SRT501 dataset comparison with 50, 39, 6 and 11 supporting state changes respectively (Figures 2[b] and 3[a]). Ppargc1α, Pparα and Pparγ also have reported roles in transcriptional regulation of mitochondrial biogenesis [35-37]. The increased metabolism and mitochondrial biogenesis network in Figure 3[a] also includes increased transcriptional activity of Esrrα which can upregulate mitochondrial respiration genes [38,39]. Finally, Nrip1 (RIP140) is a known transcriptional repressor of genes involved mitochondrial biogenesis and a corepressor of Esrrα and Pparα, and the reduced transcriptional activity of Nrip1 hypothesis is supported by 13 state changes (for a review[40]).

To determine if the hypotheses underlying the increased metabolism and mitochondrial biogenesis network are supported in the SRT1720 dataset comparison, we examined the probe sets for the state changes supporting the increased **t**ranscriptional **a**ctivity **of **Pparα (taof(Pparα)) and increased **t**ranscriptional **a**ctivity **of **Ppargc1α (taof(Ppargc1α)) hypotheses to determine whether their directionality upon SRT1720 treatment was conserved. Increased taof(Pparα) is the most statistically significant hypothesis in Figure 3[a], supported by 50 state changes and all 75 probe sets mapping to these 50 state changes in the SRT501 dataset comparison are modulated in the same direction after SRT1720 treatment (p < 10^-5^, Figure 3[b]). Similarly, for the 6 state changes supporting increased taof(Ppargc1α) in the SRT501 dataset comparison, we determined that all 16 probes mapping to these state changes were modulated in the same direction after SRT1720 treatment (p < 10^-5^, Figure 3[c]). Finally, we observed conserved directionality between the SRT1720 and SRT501 dataset comparisons for the state changes supporting increased taof(Pparγ), increased taof(Pparδ), increased taof(Esrrα) and decreased taof(Nrip1) (see Additional file 2). Taken together these data suggest that SRT1720, like SRT501, increases transcriptional activities of Ppargc1α and the other Ppar family members. Ultimately, these molecular signaling events contribute, at least in part, to efficacy of SRT501 and SRT1720 in improving glucose homeostasis and insulin sensitivity in animal models of T2DM (Figure 2[d]).

Although, Figure 3[a] predicts activation of Ppar family members as a molecular signaling component downstream of SIRT1 activation, neither SRT501 nor SRT1720 recapitulates key pharmacological activities of known Ppar agonists, fibrates (Ppara) and thiazolidinediones (Pparg). In animals dosed with SRT501 or SRT1720, there were no increases in molecular markers of peroxisomes in liver nor were there increases in liver mass (data not shown), as one would observe in animals treated with the fibrate class of drugs. There also were no observed side-effects indicative of thiazolidinedione treatment, such as edema, increased adipocyte proliferation and increased body weight (data not shown). Thus, while small molecule activation of SIRT1 impinges on Ppar activation at the molecular signaling level, SRT501 and SRT1720 are pharmacologically distinct from both fibrates and thiazolidinediones.

In order to further validate the predicted increase in mitochondrial biogenesis upon SRT501 treatment, which is also activated in response to SRT1720 treatment, we performed a number of *in vitro *and *in vivo *studies. It has been previously shown that resveratrol [31] and SRT1720 [32] increases citrate synthase activity, an enzymatic marker for mitochondrial content, in skeletal muscle of mice treated with these compounds. To demonstrate that these SIRT1 activators can directly increase mitochondrial content in a cell based model, we measured CS activity in C2C12 myotubes treated with vehicle, SRT501 or SRT1720 for 48 hours. As shown in Figure 3[d], both SRT501 (12.5 and 25 μM) and SRT1720 (2.5 μM) induced statistically significant increases in CS activity, suggesting increased mitochondrial content in these cells. Finally, we measured ATP, an indirect marker for mitochondrial energy output, in NCI-H358 cells treated with SRT501 (50 μM) or SRT1720 (1 μM) for 48 hours. Figure 3[a] demonstrates that both SRT501 and SRT1720 increase ATP levels with statistical significance compared to vehicle. In conclusion, these data, combined with previous reports on resveratrol and SRT1720, strongly support the network shown in Figure 3[a], in which small molecule activators of SIRT1 increase mitochondrial biogenesis.

### SRT501 and SRT1720 blunt inflammatory signaling pathways

**Figure 4:**
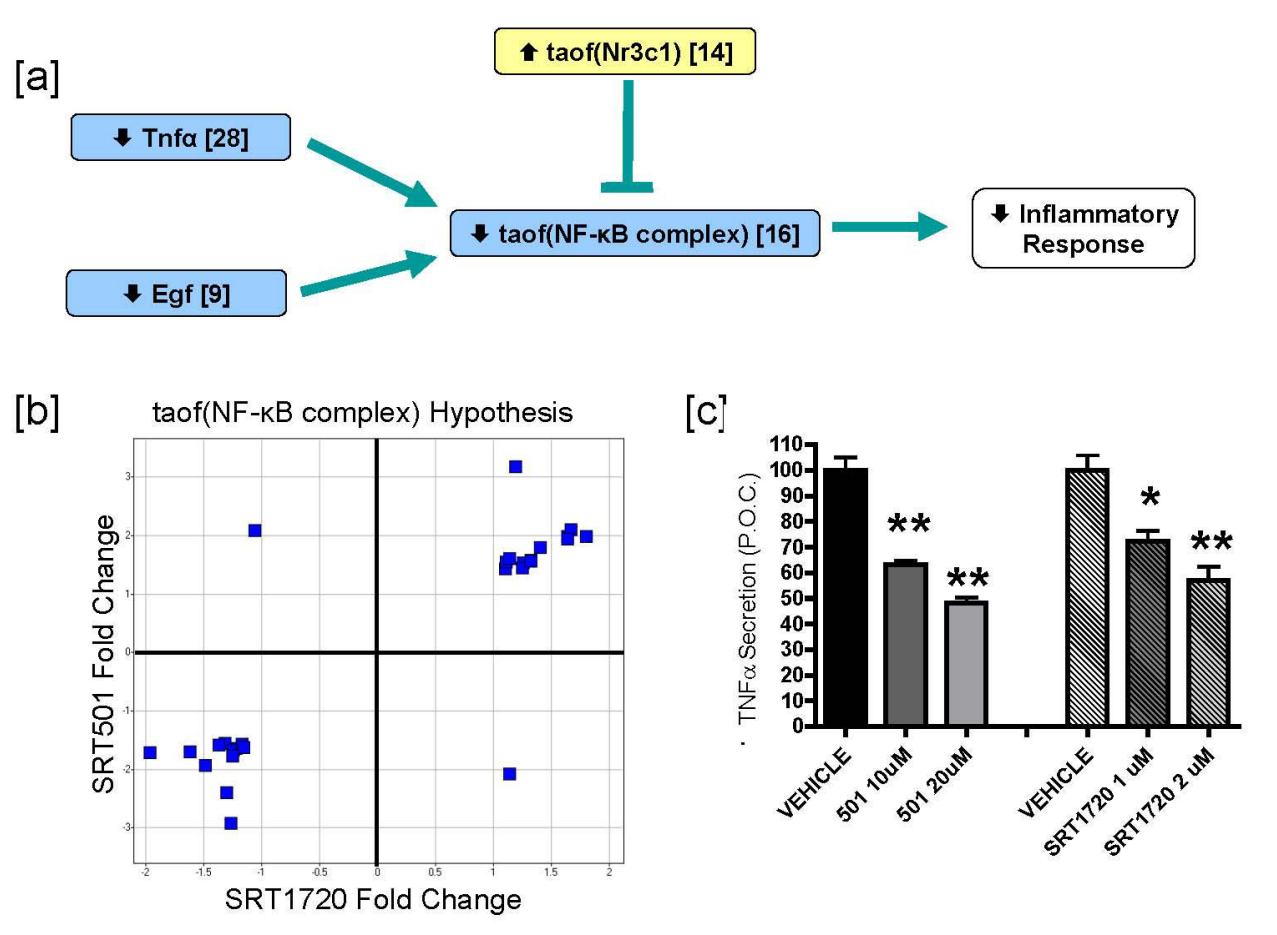
**SRT501 and SRT1720 treatment leads to decreased inflammatory signaling**. [a] Flow diagram of a network depicting statistically significant hypotheses supporting decreased pro-inflammatory signaling in DIO mouse livers after 3 days of treatment. Protein names lacking the taof prefix indicate decreased abundance, e.g. decreased abundance of Tnfα ligand leads to decreased taof(NF-κB complex). [b] Scatter plot obtained by graphing fold changes of significant probe sets supporting the decreased taof(NF-κB complex) hypothesis in the SRT1720 dataset comparison versus the SRT501 dataset comparison. [c] RAW 264.7 macrophages were treated with vehicle (0.2% DMSO), SRT501 (10 and 20 μM) or SRT1720 (1 and 2 μM) for 1 hour. Subsequently, TNFα secretion was induced by addition of LPS at 100 ng/ml for 1 hour. Cell Supernatants were collected and TNFα levels were measured by ELISA. TNFα levels are depicted as a percent of vehicle control (P.O.C.). P values were measured using an unpaired, 2-tailed t test. n = 3 replicates per group for each compound treatment. n = 8 replicates per group for each vehicle treatment. Error bars represent standard error of the mean. (*p < 0.05, **p < 0.005).

Another well characterized hallmark of CR is decreased inflammation [3,41-45]. Tnfα and Egf are known inducers of NF-κB complex transcriptional activity, and Nr3c1 (GR) represses NF-κB complex-induced transcription by a direct binding event as well through indirect mechanisms such as transactivation of the IκB gene (reviewed in[46]). Reverse causal analysis identified these inflammatory mediators as statistically significant hypotheses after SRT501 treatment. Figure 4[a] depicts the decreased inflammation network with the following support: decreased Tnfα protein abundance (supported by 28 state changes), decreased Egf protein abundance (supported by 9 state changes), decreased transcriptional activity of the NF-κB complex (supported by 16 state changes) and increased transcriptional activity of Nr3c1 (supported by 14 state changes). In this model, decreased NF-κB complex transcriptional activity is supported by decreased Tnfα and Egf abundance, as well as increased Nr3c1 activity. In order to determine whether the state changes consistent with decreased inflammatory signaling were observed in the SRT1720 dataset comparison, we examined the 27 probe sets that map to the 16 state changes supporting the decreased taof(NF-κB complex) hypothesis and determined that 25 of 27 probe sets were modulated in the same direction between the SRT501 and SRT1720 dataset comparisons (p < 10^-5^, Figure 4[b]). Similar analyses were performed for the other three hypotheses in Figure 4[a] (decreased Tnfα, decreased Egf and increased taof(Nr3c1), supplementary data), and it was determined that in each case, the underlying changes in gene expression were consistently modulated in the same direction.

Next, we examined whether SRT501 and SRT1720 could affect inflammatory signaling in an *in vitro *cell based model, LPS-induced TNFα secretion in RAW264.7 macrophages. Cells were pre-incubated with compounds at the indicated doses for 1 hour and then were treated with 100 ng/ml LPS for an additional hour. TNFα concentrations in cell supernatants were then measured by ELISA. Figure 4[c] shows that SRT501 (20 μM) and SRT1720 (2 μM) inhibit TNFα secretion by ~50% and ~40% respectively. Taken together, these findings support the hypothesis that SRT501 and SRT1720 inhibit inflammation and are consistent with prior reports suggesting that SIRT1 may inhibit inflammatory signaling pathways [47-52].

## Discussion

We have previously described the identification and characterization of small molecule activators of SIRT1. These compounds increase SIRT1 enzymatic activity by lowering the K_m _of SIRT1 for its acetylated substrates. Importantly, these drug-like molecules are efficacious in improving glucose and insulin homeostasis in multiple animal models of insulin resistance and T2DM [31,32]. In the present study, we characterize the early molecular signaling mechanisms potentially underlying the therapeutic efficacy of SIRT1 activation by small molecules in a diet induced obesity model. To this end we employed a combination of Causal Network Modeling of transcriptional profiling data and supporting *in vivo *and *in vitro *data.

Reverse causal analysis identified CR as a statistically significant hypothesis in the SRT501 dataset comparison. To determine whether this hypothesis is also supported in the SRT1720 dataset comparison, we analyzed the underlying state changes at the probe set level. In doing so we determined that all 73 probe sets covering 54 changes in gene expression displayed conserved directionality between the two dataset comparisons (p < 10^-5^). Hence, these two chemically distinct small molecule activators of SIRT1 recapitulate many of the molecular events of calorie restriction *in vivo *and importantly work via the same molecular mechanism *in vivo *– SIRT1 activation, a conclusion which is supported previous studies which have shown that SRT501-resveratrol and SRT1720 increase the deacetylation of SIRT1 substrates (e.g. p53, Ppargc1a, and Foxo1a) *in vivo *and *in vitro*. Additionally, previous studies have demonstrated that SRT501[30,31,53] and SRT1720[32,54], like calorie restriction [4,8], enhance oxidative metabolism, protect against diet induced obesity, increase exercise endurance, and improve glucose and insulin homeostasis in rodent models of metabolic disease. These findings are consistent with the role of SIRT1 downstream of calorie restriction, which, as a therapeutic strategy, has proven efficacious in improving the metabolic profiles in rodents, large mammals, monkeys and humans [3-5,55].

Reverse causal analysis also identified increased metabolism, mediated primarily by transcriptional activation of Ppar family members, as a network supported by six statistically significant hypotheses in the SRT501 dataset comparison. Furthermore, we determined that the changes in gene expression which support these hypotheses are modulated in the same direction in the SRT501 and SRT1720 treatment groups. Ppar-mediated signaling is a well documented molecular event downstream of calorie restriction [56,57]. These nuclear hormone receptors regulate a multitude of physiological pathways including lipid metabolism, glucose homeostasis, inflammation and development [37,58-60]. Notably, PPARα and PPARγ are respectively the molecular targets of the fibrate and thiazolidinedione (TZD) classes of drugs [61,62]. Fibrates are commonly used for therapeutic intervention of hypertension, atherosclerosis, and dyslipidemia. TZDs, on-the-other-hand, are insulin-sensitizing reagents frequently used in the treatment of T2DM and Metabolic Syndrome. It is important to note that, while reverse causal analysis identified Ppar activation as a hypothesis downstream of small molecule activation of SIRT1, we did not observe the full range of pharmacological effects associated with fibrate or TZD treatment, such as increased peroxisome mass, body weight gain, increased adipocyte proliferation and edema. We speculate that the levels of Ppar activation and/or the subset of upregulated Ppara gene-targets generated by treatment with SRT501 or SRT1720 are likely more consistent with those observed downstream of calorie restriction.

The exact mechanism by which SRT501 and SRT1720 increase transcriptional activation of Ppars is an area for future investigation. However, it is likely due, at least in part, to deacetylation and transcriptional activation of Ppargc1α, a phenomenon supported by transcriptional data in this study. Ppargc1α has been well characterized as a co-activator for numerous nuclear hormone receptors including Ppars [36]. Also, Ppargc1α is known to be deacetylated at multiple lysine residues by SIRT1, leading to increased Ppargc1α transcriptional activity [19].

Importantly, the effect of SIRT1 activation on Ppar activity is likely tissue-specific and family member-specific. For example, previous work in rodents has demonstrated that SIRT1 represses Pparγ mediated transcription, leading to increased fat mobilization and inhibition of adipogenesis [63]. Future studies may elucidate whether Ppar activation in tissues other than liver is important for the efficacy of SIRT1 activating compounds and what the relative contribution of specific Ppar family members is in a tissue-specific context.

Closely linked to the increased metabolism and Ppar family activity is increased mitochondrial biogenesis, a phenomenon strongly associated with CR [64]. The key node of this network is transcriptional activation of Ppargc1α, which, as mentioned previously, is deacetylated by SIRT1, leading to increased transcriptional activity of Ppargc1α [19]. This network is also supported by the increased transcriptional activities of Pparα, Pparγ and Essrα and by decreased transcriptional activity of Nrip1. Pparα, Pparγ and Essrα can be coactivated by Ppargc1α, leading to increased expression of target genes, many of which are involved in mitochondrial respiration and biogenesis [37,65]. Conversely, Nrip1 (RIP140) acts a transcriptional co-repressor of several nuclear hormone receptors, including Esrrα, leading to decreased expression of genes involved in oxidative metabolism and mitochondrial biogenesis [40].

Within the context of the liver, the increased mitochondrial biogenesis may contribute to an improved metabolic profile via increased efficiency in fatty acid oxidation and lowering of serum triglycerides, both of which have been shown to improve insulin sensitivity in peripheral tissues [66]. Furthermore, it is certainly plausible that increased mitochondrial biogenesis in other tissues, such as skeletal muscle and adipose tissue, also contributes to therapeutic efficacy of SIRT1 activating compounds. In fact, previous reports have indicated that both resveratrol and SRT1720 increase mitochondrial function in skeletal muscle [31,32]. This increase in mitochondrial capacity allows for efficient substrate switching between lipids, during times of fasting, and carbohydrates in response to insulin [67,68]. Moreover, mitochondrial oxidative capacity in skeletal muscle has been shown to be a strong predictor of insulin sensitivity in humans [69].

Finally, SRT501 and SRT1720 mimic calorie restriction as demonstrated by the hypotheses consistent with decreased inflammation. As previously mentioned, calorie restriction is well known to inhibit inflammatory processes. This hypothesis is based upon decreases in signaling of Tnfα, Egf and NF-κB complex and upon increased transcriptional activity of the glucocorticoid receptor, Nr3c1. Tnfα is a well characterized inflammatory cytokine and has been implicated in the pathogenesis of numerous diseases, including autoimmune disorders, cancer, insulin resistance and Metabolic Syndrome [70-72]. Similarly, the NF-κB complex, which lies downstream of the Tnfα signaling cascade, is a transcriptional complex, which regulates the transcription of wide variety of target genes including inflammatory signaling factors, such as Tnfα [73]. Egf also has an established role in inflammatory signaling, most notably implicated as a fibrogenic factor underlying the pathogenesis of COPD, cystic fibrosis and cardiovascular disease [74,75]. Finally, the glucocorticoid receptor Nr3c1 is a central regulator of inflammatory responses, capable of transcriptionally repressing pro-inflammatory genes and of transcriptionally activating anti-inflammatory genes (reviewed in [76]). In summary, the predicted decrease in inflammation is comprised of a combination of upregulation of anti-inflammatory pathways and downregulation of pro-inflammatory pathways. Elucidation of the exact mechanism by which SIRT1 activation leads to these events will be an area for future investigation.

Notably, this network is supported by *in vitro *data, which demonstrates that both SRT501 and SRT1720 induce dose-dependent inhibition of LPS-stimulated Tnfα secretion in a cultured macrophage cell line. Also, several prior reports have implicated SIRT1 [47-49,51,77] and resveratrol [78-80] as negative regulators of inflammation both *in vitro *and *in vivo*.

The decreased inflammation network supports the calorie restriction model, as CR has been shown to inhibit inflammation in multiple animal models, including humans. Whether this decreased inflammation contributes to the therapeutic efficacy of our SIRT1 activating compounds remains under investigation. Certainly, there is increasing evidence that the development of insulin resistance in obese individuals involves paracrine interplay between white adipose tissue and infiltrating macrophages. At the molecular level, this pathogenic progression is characterized by an increase in secretion of inflammatory cytokines, such as TNFα, IL-6 and MCP-1, and a decrease of anti-inflammatory adipokines, such as adiponectin (reviewed in[81]). Exacerbation of this pathogenic inflammatory response to include tissues such as the liver and vasculature can ultimately contribute to the onset of T2DM and/or Metabolic Syndrome. It has been reported that the anti-inflammatory effects of TZDs, mediated through PPARγ, are essential for the therapeutic efficacy of that drug class in treating T2DM [81]. Hence, it is quite plausible that SIRT1 activating compounds improve glucose and insulin homeostasis, in part, by inhibiting inflammatory signaling.

## Conclusion

In conclusion, our modeling effort provides evidence that SIRT1 activating compounds mimic many of the benefits of calorie restriction *in vivo*. These compounds increase metabolism and mitochondrial biogenesis, primarily through transcriptional activation of Ppargc1α and Ppar family members. Additionally, these molecules decrease inflammatory signaling, which could theoretically improve cytokine and adipokine profiles and reduce macrophage burden in key target tissues. The total effect of such phenomena could result in increased insulin sensitivity in multiple tissues, including liver, muscle and fat. Additionally, these compounds enhance glucose homeostasis and abrogate the diabetic phenotype in multiple animal models of T2DM. Although calorie restriction has been characterized as an effective strategy in the treatment of diseases of aging, such as T2DM, it is logistically very difficult to adhere to such a regimen due to the hunger and pleasure drives for food intake. Hence, we speculate that SIRT1 activating compounds, currently in clinical trials for multiple indications, could be a promising alternative to CR in combating these diseases.

## Methods

### Cell culture

RAW 264.7 macrophages (ATCC #TIB-71) were maintained in DMEM (Invitrogen #11995) with 10% Fetal Bovine Serum (low endotoxin; Benchmark; Gemini #100-106). C2C12 (ATCC #CRL-1772) were maintained in DMEM (Invitrogen #11995) with 10% Fetal Bovine Serum (Foundation; Gemini #900-108). NCI-H358 cells were maintained in DMEM (Invitrogen #11995) with 10% Fetal Bovine Serum (Invitrogen). All cell lines were cultured in the presence of penicillin/streptomycin (Invitrogen).

### ATP assay

NCI-H358 cells were plated at 10^4 ^cells per well in 96 well plates. 16 hours after plating cells were treated with either SRT501 (50 μM), SRT1720 (1 μM), or vehicle control (DMSO, 0.5% final concentration). All compounds were delivered at a final DMSO concentration of 0.5%. After 48 hours of incubation with compounds under normal growth conditions, cells viability was assessed using AlamarBlue™ reagent (Invitrogen). Cells were grown with viability reagent for 2 hours under normal growth conditions. AlamarBlue™ signal was measured using a SpectraMax M5 plate reader (Molecular Dynamics; excitation 545 nm; emission 575 nm). Cell supernatant was then removed and cells were washed 2 times in PBS. ATP levels were measured using the ATPLite 1Step kit (Perkin Elmer), following the manufacturers protocol. Bioluminescence was measured using a SpectraMax M5 plate reader.

### Citrate synthase assay

Citrate Synthase enzymatic activity was measured using a modified version of the protocol described by Moyes, 1997[82]. C2C12 were seeded in 96 well plates and grown to confluence. Subsequently, cell media was changed to differention media: DMEM (Invitrogen #11995) containing 2% Horse serum (Hyclone #SH30074.02) and penicillin/streptomycin (Invitrogen). After two days of differentiation, cells were treated with fresh differentiation media containing containing one of the following, SRT501 (12.5 or 25.0 μM), SRT1720 (1.25 or 2.50 μM), or vehicle control (DMSO, 0.5% final concentration). All compounds were delivered at a final DMSO concentration of 0.5%. After 48 hours of incubation with compounds under differentiation conditions, media was then removed and cells were washed 2 times in PBS. Cells were lysed for 15 minutes at 4°C, using an addition of 90 μl of Cell Lytic M^® ^(Sigma) to each well. Next, 100 μl of 2× Reaction Buffer (0.1 M Tris HCl pH 8.0, 0.2 mM 5,5'-dithio-bis(2-nitrobenzoic acid), 0.6 mM Acetyl Coenzyme A) was added to each well. Citrate Synthase reaction were initiated by addition of 10 μl of 10 mM oxaloacetate to well. Final concentrations of reaction mixture components were 50 mM Tris HCl pH 8.0, 0.1 mM 5,5'-dithio-bis(2-nitrobenzoic acid), 0.3 mM Acetyl Coenzyme A, and 0.5 mM oxaloacetate. After addition of oxaloacetate, plates were shaken for 30 seconds and then read, using a SpectraMax M5 plate reader (Absorbance 412 nm). Absorbance readings were taken every 30 seconds for 15 minutes. Raw Citrate Synthase activity was expressed as the slope of the linear regression plot of the change in Abs 412 nm over time. Cell viability was measured as described for ATP Assay.

### TNFα secretion assay

RAW 264.7 macrophages were seeded at 4 × 10^4 ^cells per well in 96 well plates. 16 hours after seeding cells were treated with either SRT501 (10 or 20 μM), SRT1720 (1 or 2 μM), or vehicle (DMSO; 0.2% final concentration). All compounds were delivered at a final DMSO concentration of 0.2%. After 1 hour of incubation with compound under normal growth conditions, 100 ng/ml (final concentration) of lippopolysaccharide (*E. coli*; Calbiochem) or vehicle control (water) was added to each well. Following 1 hour of incubation with LPS (to stimulate TNFα secretion), cell supernatants were removed from each well and transferred to TNFα ELISA plates (mouse-specific, Invitrogen). TNFα levels were measured according to the manufacturer's protocol. ELISA signal was measured by reading Absorbance at 450 nm using a SpectraMax M5 plate reader. Cell viability was measured as described for ATP Assay.

### Diet induced obesity model

This study was approved by the Institutional Animal Care and Use Committee (IACUC) of Enanta (Watertown, MA) (Protocol # 2005-17), and carried out in accordance with federal (USA) IACUC regulations.

Nine week old C57BL/6 male mice (Charles River Labs) were fed a high fat diet (60% calories from fat; Research Diets) until their mean body weight reached approximately 40 g. The mice were then divided into test groups (6-10 per group). SRT1720 (100 mg/kg) and SRT501 (500 mg/kg) were administered once daily via oral gavage. The vehicle used was 2% HPMC + 0.2% DOSS. Individual mouse body weights were measured twice weekly. Fasted blood glucose was measured after 3 weeks of dosing with compounds. To obtain fasting blood glucose measurements, food was removed late in the day (4 PM) before the glucose reading was taken the following morning.

Fed Insulin levels were measured from plasma after 4 weeks of dosing with SRT501 or vehicle and after 6 weeks of dosing with SRT1720 or vehicle. Blood insulin levels were measured from Linco EZ-RMI13K. To obtain fed insulin measurements, food was removed one hour before the blood collection.

### Transcriptional profiling

Livers were harvested after 3 days of dosing (1–2 hours after the third dose) with compounds or control (4 mice per group). Livers were snap-frozen on liquid nitrogen and shipped to Expression Analysis, Inc. (Durham, NC) for RNA preparation and transcriptional profiling. RNA was purified using the RNeasy Mini Kit (QIAGEN) according to the manufacturer's procedure, including the on-column DNase I digestion. For expression profiling, 2 μg of total RNA was converted to double stranded cDNA using the One-Cycle cDNA Synthesis Kit from Affymetrix (Part Number 900431) and in vitro transcribed using the IVT Labeling Kit from Affymetrix (Part Number 900449) according to Expression Analysis' standard operating procedures. Biotin-labeled cRNA at a final concentration of 50 ng/μl was hybridized to Mouse Genome 430 2.0 Arrays according to the manufacturer's procedure.

### Selection of molecular state changes for analysis

RNA expression data were generated using Affymetrix Mouse Genome 430 2.0 expression microarrays and analyzed using the "affy" and "limma" packages of the Bioconductor suite of microarray analysis tools available for the R statistical environment [83-86]. RMA background correction and quantile normalization was used to generate microarray expression values. An overall linear model was fit to the data for all sample groups and specific contrasts of interest were evaluated to generate raw p-values for each probe set on the expression array [87]. The Benjamini-Hochberg FDR method was then used to correct for multiple testing effects. Probe sets were considered to have changed qualitatively in a specific comparison if an adjusted P-value of 0.05 was obtained, both their average expression intensity was above 250 and they had a fold change greater than 1.3. Alternatively, if the average intensity in each dataset was below 250 but they had a fold change greater than 1.5 and were present by Affymetrix absence/presence calls in one of the datasets, probe sets were also considered changed. Genes represented by multiple probe sets were considered to have changed if at least one probe set was observed to change. Expression changes that met these criteria are called statistically significant "RNA state changes" and have the directional qualities of "up" or "down", i.e., they can be upregulated or downregulated in response to compound treatment.

### Knowledgebase and mouse knowledge assembly model

The substrate for analysis of RNA state changes observed in the SRT501 dataset comparison is the mouse knowledge assembly model, which is derived from a global Genstruct knowledgebase. This knowledgebase is a collection of biological concepts and entities and their causal relationships and is derived from peer-reviewed scientific literature as well as other public and proprietary databases and has been constructed during the course of projects in areas such as inflammation, metabolic diseases, cardiovascular injury, liver injury and cancer. The mouse knowledge assembly model is the set of mouse-specific causal assertions that has been augmented with orthologous causal assertions derived from either rat or human sources and is competent for reverse causal analysis. An example causal assertion would be increased transcriptional activity of NF-κB complex causing an increase in the expression of insulin response factor 1(Irf1) [88]. Each such causal assertion has a specific scientific citation, and the assembled collection of these causal assertions is referred to as the mouse knowledge assembly model in this paper.

### Reverse causal analysis: automated hypothesis generation

In the case of RNA expression data, Causal Network Modeling employs an automated reverse causal analysis approach which interrogates the mouse knowledge assembly model to identify upstream controllers for the RNA state changes observed in the experiment. These upstream controllers are called hypotheses as they are statistically significant potential explanations of the RNA state changes. Hypothesis generation is performed automatically by a computer program that utilizes the mouse knowledge assembly model to identify hypotheses that explain the input RNA state changes, prioritized by multiple statistical criteria. Each hypothesis is scored according to two probabilistic scoring metrics, richness and concordance, which examine distinct aspects of the probability of a hypothetical cause explaining a given number of RNA state changes. Richness is the probability that the number of observed RNA state changes connected to a given hypothesis could have occurred by chance alone. Concordance is the probability that the number of observed RNA state changes that match the directionality of the hypothesis (e.g., increased or decreased kinase activity for a kinase, increased or decreased transcriptional activity for a transcription factor, etc.) could have occurred by chance alone. A hypothesis is considered to be statistically (although not necessarily biologically) significant if it met richness and concordance cutoffs of 0.1. The statistical threshold of 0.1 has been determined as an appropriate cutoff to begin building networks; known biology downstream of a given perturbation has been appropriately modeled using these thresholds. Automated reverse causal analysis on the SRT501 dataset comparison yielded 146 statistically significant hypotheses, which were further investigated and prioritized by evaluation of their biological relevance to the experimental context, whether they are causally linked to phenotypes and processes relevant to known sirtuin function in the literature and if they are causally downstream of Sirt1 activation. To build networks, core hypotheses which are strongly statistically significant (< 0.05) are first identified, then other hypotheses which fall in the 0.05 to 0.1 range are used as additional support. Using both the information stored in the mouse knowledge assembly model and from literature investigation, causal links between selected hypotheses were identified enabling incorporation into an overall biological network capable of explaining the effects of SRT501 treatment, called the Causal Network Model (CNM). Out of the 146 hypotheses identified by reverse causal analysis, twelve hypotheses make up the three pathways described in the manuscript. These pathways were chosen because to date, they have been validated by *in vitro *and *in vitro *experimental data.

### Comparing the SRT501 causal network model to the SRT1720 dataset comparison

In order to compare the hypotheses identified based on SRT501 treatment to the SRT1720 dataset comparison, we compared the directionality of the RNA state changes supporting statistically significant hypotheses in the SRT501 dataset comparison to those in the SRT1720 dataset comparison on the probe set level. The number of probe sets with fold changes in the same direction in the SRT501 and SRT1720 dataset comparisons was determined for each of the twelve hypotheses discussed in this paper. P-values were calculated based on binomial distribution assuming no relationship between the SRT501 and SRT1720 dataset comparisons. This method allowed us to detect whether general trends in the directionality of the RNA state changes supporting hypotheses identified based on SRT501 treatment were also present after SRT1720 treatment.

## Authors' contributions

OB contributed to the study design, carried out the tissue harvest, and participated in the data interpretation. RDK led causal modeling efforts, aided in study design, and participated in the data interpretation. DJG led animal studies and tissue collection. BPF contributed to causal modeling efforts and data interpretation. WL led statistical analysis of data. HLG, GR, AVL, and DPC examined the effect of compounds on mitochondrial biogenesis in cell based assays. KI and JS conducted cell based TNFα release assays. RJJ contributed to causal modeling efforts. SL and AI aided animal studies. PJE, PDL, KOE, MRJ, and JCM aided in study design, data interpretation, and in editing of manuscript. JJS headed drafting of manuscript, led data interpretation efforts and study design.

## Supplementary Material

Additional file 1 **Causal network model knowledgebase.** This file contains the knowledge used in this CNM effort. Click here for file

Additional file 2 **Supplemental data and legends.** Supplemental data and legends contains figures detailing state changes underlying many key nodes that were identified in by CNM but were not described in the main text. Click here for file
