# Supplementary material for: Small molecule activators of SIRT1 replicate signaling pathways triggered by calorie restriction in vivo
Source: BMC Syst Biol. 2009 Mar 10;3:31. doi: 10.1186/1752-0509-3-31 (PMC2660283; doi:10.1186/1752-0509-3-31)
Supplement: Additional file 2 — Supplemental data and legends. Supplemental data and legends contains figures detailing state changes underlying many key nodes that were identified in by CNM but were not described in the main text. [file 1752-0509-3-31-S2.pdf]

Supplemental data figure legends

Supplemental Data Figure 1 -State changes supporting statistically significant hypotheses described in the manuscript.

Green – measured and observed increase in RNA expression. Red – measured and observed decrease in RNA expression. Yellow – hypothesized increase in abundance or activity. Blue – hypothesized decrease in abundance or activity.

[a] Increased CR hypothesis

[b] Resveratrol hypothesis

[c] Increased transcriptional activity of Esrrα hypothesis

[d] Decreased transcriptional activity of Nrip1 hypothesis

[e] Increased transcriptional activity of Pparα hypothesis

[f] Increased transcriptional activity of Pparγ hypothesis

[g] Increased transcriptional activity of Pparδ hypothesis

[h] Increased transcriptional activity of Ppargc1α hypothesis

[i] Decreased abundance of Egf

[j] Decreased transcriptional activity of the NF-κB complex

[k] Increased transcriptional activity of Nr3c1

[l] Decreased abundance of Tnfα

Supplemental Data Figure 2 -Effects of three days of SRT1720 treatment on transcript data from mouse livers, compared to SRT501 treatment.

Scatter plot obtained by graphing fold changes of significant probe sets supporting hypotheses in the SRT1720 dataset comparison versus the SRT501 dataset comparison.

[a] Increased CR hypothesis

[b] Resveratrol hypothesis

[c] Increased transcriptional activity of Esrrα hypothesis

[d] Decreased transcriptional activity of Nrip1 hypothesis

[e] Increased transcriptional activity of Pparα hypothesis

[f] Increased transcriptional activity of Pparγ hypothesis

[g] Increased transcriptional activity of Pparδ hypothesis

[h] Increased transcriptional activity of Ppargc1α hypothesis

[i] Decreased abundance of Egf

[j] Decreased transcriptional activity of the NF-κB complex

[k] Increased transcriptional activity of Nr3c1

[l] Decreased abundance of Tnfα

Supplemental Data Figure 1

[a]

Calorie Restriction (CR)	SRT501 Dataset Comparison
Calorie Restriction (CR) hypothesis	↑
exp(1110067D22Rik)	↓
exp(4933429D07Rik)	↑
exp(Acaa1b)	↑
exp(Acadm)	↑
exp(Acot1)	↑
exp(Acot2)	↑
exp(Acsl1)	↑
exp(Acsm3)	↑
exp(Akp2)	↑
exp(Aldh9a1)	↑
exp(Angptl4)	↑
exp(Anxa7)	↑
exp(Aox1)	↑
exp(AU018778)	↑
exp(Bnip3)	↑
exp(C230093N12Rik)	↑
exp(Cd36)	↑
exp(Ceacam1)	↓
exp(Ces3)	↑
exp(Cml1)	↓
exp(Coq10b)	↑
exp(Cpt1a)	↑
exp(Cpt2)	↑
exp(Crat)	↑
exp(Creg1)	↑
exp(Ctse)	↓
exp(Cyp4a10)	↑
exp(Cyp4a14)	↑
exp(Dci)	↑
exp(Dnajb6)	↑
exp(Ehhadh)	↑
exp(Fcgrt)	↑
exp(Foxo3a)	↑
exp(Gstt2)	↑
exp(Hsd17b11)	↑
exp(Hsdl2)	↑
exp(Id2)	↓
exp(Ly6e)	↓
exp(Mbd1)	↑
exp(Nab2)	↓
exp(Oprs1)	↓
exp(Peci)	↑
exp(Pex11a)	↑
exp(Pfkfb3)	↑
exp(Sgpl1)	↓
exp(Slc22a5)	↑
exp(Slc25a20)	↑
exp(Sorbs1)	↑
exp(St3gal5)	↑
exp(Txnip)	↑
exp(Ubb)	↑
exp(Usp2)	↑
exp(Wee1)	↑
exp(Zfp306)	↑

[b]

Resveratrol Treatment	SRT501 Dataset Comparison
Resveratrol Treatment hypothesis	↑
exp(2410014A08Rik)	↑
exp(Acaa1a)	↑
exp(Acad11)	↑
exp(Acadm)	↑
exp(Acat1)	↑
exp(Acox1)	↑
exp(Acpp)	↓
exp(Acsl1)	↑
exp(Acsm3)	↑
exp(Adfp)	↑
exp(Aldh1a7)	↑
exp(Angptl4)	↑
exp(Aptx)	↑
exp(Ar)	↑
exp(Ass1)	↓
exp(Bbc3)	↓
exp(Bnip3)	↑
exp(Canx)	↑
exp(Cd36)	↑
exp(Cetn2)	↑
exp(Cks2)	↓
exp(Copz2)	↑
exp(Cpt1a)	↑
exp(Ctsl)	↓
exp(Cyp1a1)	↑
exp(Cyp1a2)	↑
exp(Enc1)	↑
exp(Fcgrt)	↑
exp(Foxo3a)	↑
exp(Gldc)	↓
exp(Hadha)	↑
exp(Hao1)	↑
exp(Hmgcs2)	↑
exp(Hspb1)	↑
exp(Id2)	↓
exp(Irf1)	↓
exp(Ly6e)	↓
exp(Maoa)	↑
exp(Mat2a)	↑
exp(Mbd1)	↑
exp(Ndrg1)	↑
exp(Nrn1)	↓
exp(Nuak2)	↓
exp(Odc1)	↑
exp(P4hb)	↑
exp(Pdia6)	↑
exp(Pdlim1)	↓
exp(Psen2)	↓
exp(Rpn2)	↑
exp(Sdc1)	↓
exp(Sirpa)	↓
exp(Slc25a20)	↑
exp(Slc29a1)	↓
exp(Slc35b1)	↑
exp(Slc3a2)	↑
exp(Slc6a6)	↓
exp(Sod2)	↑
exp(Sorbs1)	↑
exp(Tnk2)	↓
exp(Txnip)	↑
exp(Vnn1)	↑
exp(Zfp131)	↑

[c]

taof(Esrrα)	SRT501 Dataset Comparison
taof(Esrrα) hypothesis	↑
exp(Acadm)	↑
exp(Acox1)	↑
exp(Acsl1)	↑
exp(Alas1)	↑
exp(Aldh1a7)	↑
exp(Cd36)	↑
exp(Etfdh)	↑
exp(Fabp1)	↑
exp(Fabp2)	↑
exp(Hadha)	↑
exp(Hsd17b4)	↑

[d]

taof(Nrip1)	SRT501 Dataset Comparison
taof(Nrip1) hypothesis	↓
exp(Acaa2)	↑
exp(Acadl)	↑
exp(Acadm)	↑
exp(Cpt2)	↑
exp(Crat)	↑
exp(Dci)	↑
exp(Decr1)	↑
exp(Ehhadh)	↑
exp(Etfdh)	↑
exp(Hadha)	↑
exp(Hadhb)	↑
exp(Slc25a20)	↑
exp(Suclg1)	↑

[e]

taof(Pparα)	SRT501 Dataset Comparison
taof(Pparα) hypothesis	↑
exp(Acaa1a)	↑
exp(Acaa1b)	↑
exp(Acaa2)	↑
exp(Acadl)	↑
exp(Acadm)	↑
exp(Acat1)	↑
exp(Acot1)	↑
exp(Acot2)	↑
exp(Acot8)	↑
exp(Acox1)	↑
exp(Acsl1)	↑
exp(Akr1d1)	↓
exp(Apoa5)	↑
exp(Asl)	↓
exp(Ass1)	↓
exp(Cd36)	↑
exp(Cps1)	↓
exp(Cpt1a)	↑
exp(Cpt2)	↑
exp(Crat)	↑
exp(Cth)	↓
exp(Cyp1a1)	↑
exp(Cyp4a10)	↑
exp(Cyp4a14)	↑
exp(Dci)	↑
exp(Decr1)	↑
exp(Decr2)	↑
exp(Dhrs4)	↑
exp(Ech1)	↑
exp(Ehhadh)	↑
exp(Ftcd)	↓
exp(Gls2)	↓
exp(Gnmt)	↓
exp(Gstt2)	↑
exp(Hadha)	↑
exp(Hadhb)	↑
exp(Hal)	↓
exp(Hmgcs2)	↑
exp(Hsd17b4)	↑
exp(Id2)	↓
exp(Mgll)	↑
exp(Odc1)	↑
exp(Pctp)	↑
exp(Peci)	↑
exp(Pex11a)	↑
exp(Pla2g6)	↑
exp(Slc22a5)	↑
exp(Slc25a20)	↑
exp(Sorbs1)	↑
exp(Vnn1)	↑

[f]

taof(Pparγ)	SRT501 Dataset Comparison
taof(Pparγ) hypothesis	↑
exp(Acaa1a)	↑
exp(Acaa1b)	↑
exp(Acadl)	↑
exp(Acadm)	↑
exp(Acat1)	↑
exp(Acox1)	↑
exp(Acsl1)	↑
exp(Adfp)	↑
exp(Angptl4)	↑
exp(Cd36)	↑
exp(Cfd)	↑
exp(Cited2)	↓
exp(Crat)	↑
exp(Ctps)	↑
exp(Cyp4a10)	↑
exp(Cyp4a14)	↑
exp(Ehhadh)	↑
exp(Etfdh)	↑
exp(Fabp1)	↑
exp(Hadha)	↑
exp(Hadhb)	↑
exp(Hmgcs2)	↑
exp(Hsd17b12)	↑
exp(Id2)	↓
exp(Maoa)	↑
exp(Mgll)	↑
exp(Ndg2)	↑
exp(Orm3)	↓
exp(Paqr7)	↑
exp(Pctp)	↑
exp(Pex11a)	↑
exp(Retsat)	↑
exp(S3-12)	↑
exp(Slc25a20)	↑
exp(Slc29a1)	↓
exp(Sorbs1)	↑
exp(Tpm2)	↑
exp(Txnip)	↑
exp(Vnn1)	↑

[g]

taof(Pparδ)	SRT501 Dataset Comparison
taof(Pparδ) hypothesis	↑
exp(Acaa1a)	↑
exp(Acadl)	↑
exp(Acox1)	↑
exp(Acsl1)	↑
exp(Adfp)	↑
exp(Cd36)	↑

[h]

taof(Ppargc1α)	SRT501 Dataset Comparison
taof(Ppargc1α) hypothesis	↑
exp(Acat1)	↑
exp(Cpt1a)	↑
exp(Slc25a20)	↑
exp(Acox1)	↑
exp(Acadm)	↑
exp(Sod2)	↑

[i]

Egf	SRT501 Dataset Comparison
Egf hypothesis	↓
exp(Acpp)	↓
exp(Ar)	↑
exp(Atp4a)	↓
exp(Cks2)	↓
exp(Cyp1a1)	↑
exp(Myo1b)	↓
exp(Ndrg1)	↑
exp(Psen2)	↓
exp(Stat5b)	↓

[j]

taof(NF-κB complex)	SRT501 Dataset Comparison
taof(NF-κB complex) hypothesis	↓
exp(Acpp)	↓
exp(Acsl1)	↑
exp(Aldh1a7)	↑
exp(Ar)	↑
exp(Ass1)	↓
exp(Bnip3)	↑
exp(Cetn2)	↑
exp(Irf1)	↓
exp(Ly6e)	↓
exp(Nuak2)	↓
exp(Pdlim1)	↓
exp(Sdc1)	↓
exp(Sirpa)	↓
exp(Slc29a1)	↓
exp(Slc6a6)	↓
exp(Tnk2)	↓

[k]

taof(Nr3c1)	SRT501 Dataset Comparison
taof(Nr3c1) hypothesis	↑
exp(Acadm)	↑
exp(Acat1)	↑
exp(Acsm3)	↑
exp(Akp2)	↑
exp(Angptl4)	↑
exp(Bbc3)	↓
exp(Ces3)	↑
exp(Cfd)	↑
exp(Cyp1a1)	↑
exp(Cyp1a2)	↑
exp(Enc1)	↑
exp(Ndrg1)	↑
exp(Pfkfb3)	↑
exp(Vdp)	↑

[l]

Tnfα	SRT501 Dataset Comparison
Tnfα hypothesis	↓
exp(Acpp)	↓
exp(Acsl1)	↑
exp(Akr1a4)	↑
exp(Aldh1a7)	↑
exp(Ass1)	↓
exp(Canx)	↑
exp(Cetn2)	↑
exp(Cfd)	↑
exp(Cpt1a)	↑
exp(Cpt2)	↑
exp(Cyp1a1)	↑
exp(Cyp1a2)	↑
exp(Dci)	↑
exp(Ech1)	↑
exp(Es22)	↑
exp(Gpiap1)	↓
exp(Irf1)	↓
exp(Ly6e)	↓
exp(Mgll)	↑
exp(Nuak2)	↓
exp(Oprs1)	↓
exp(Psen2)	↓
exp(Slc16a7)	↑
exp(Slc25a20)	↑
exp(Slc29a1)	↓
exp(Slc5a6)	↑
exp(Slc6a6)	↓
exp(Tnk2)	↓

Supplemental Data Figure 2

[a]

Calorie Restriction (CR) Hypothesis

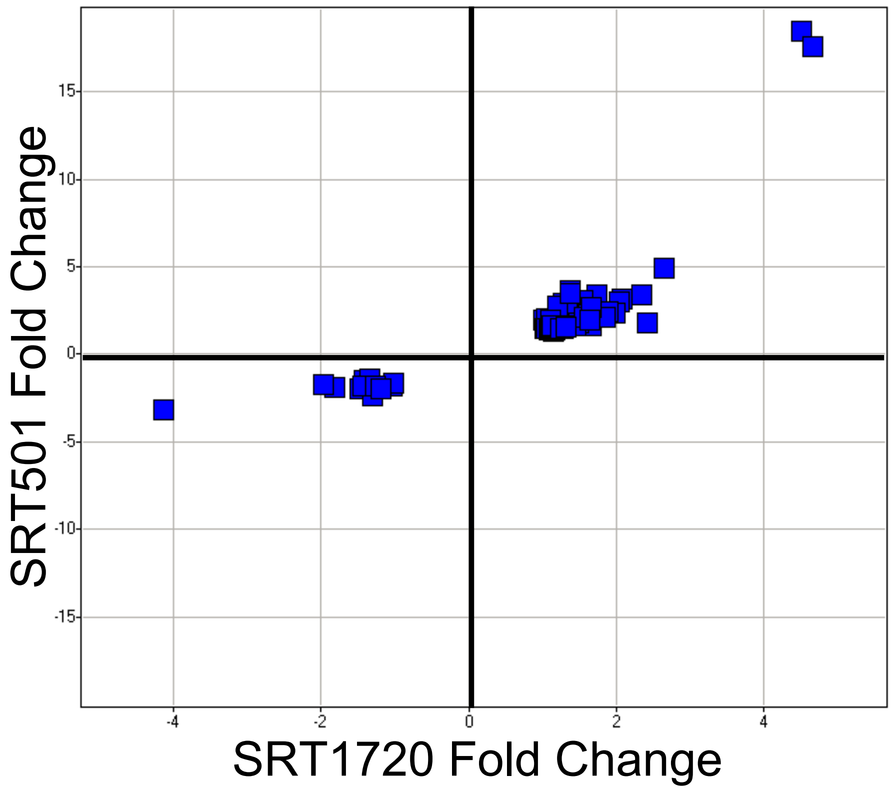

73 of 73 probe sets modulated in same direction after SRT501 and SRT1720 treatment ($P<10^{-5}$)

[b]

Resveratrol Treatment Hypothesis

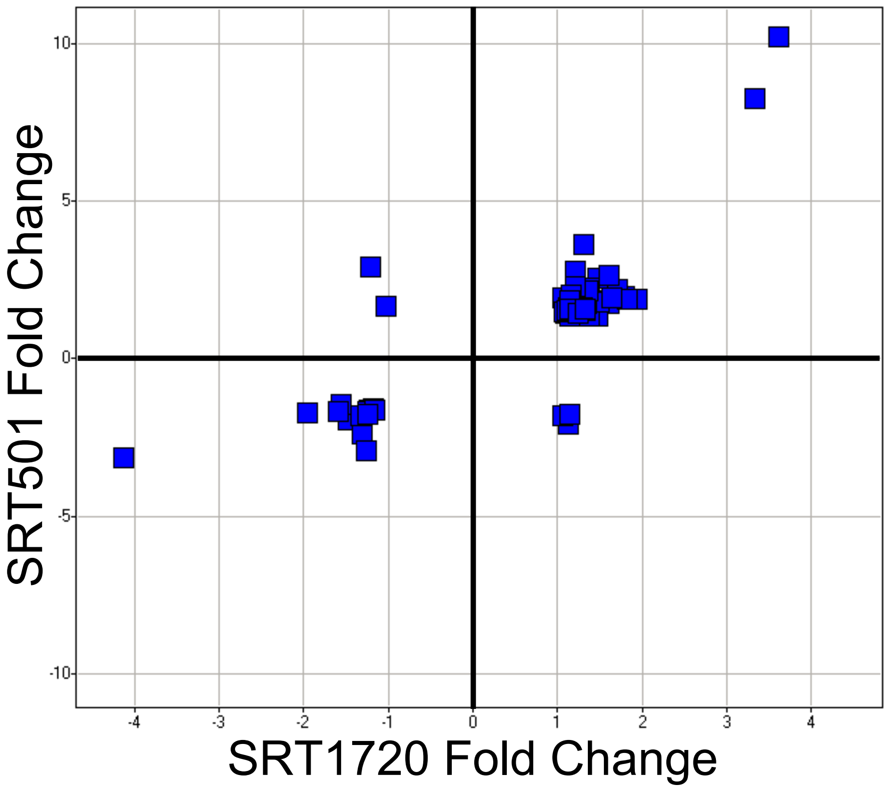

64 of 69 probe sets modulated in same direction after SRT501 and SRT1720 treatment ($P<10^{-5}$)

[c]

taof(Esrrα) Hypothesis

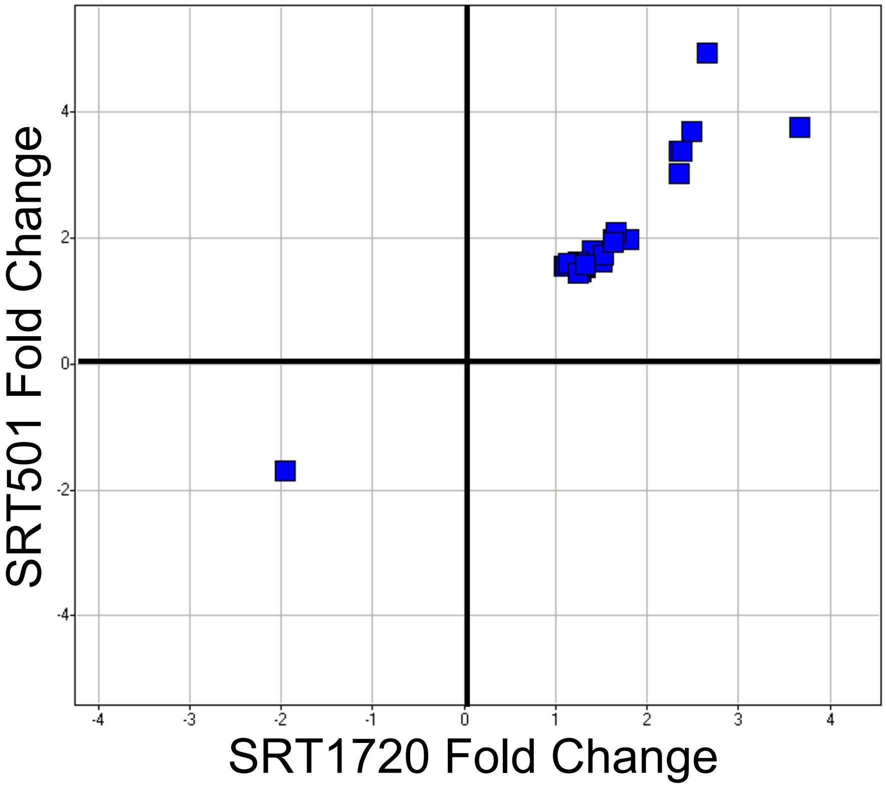

25 of 25 probe sets modulated in same direction after SRT501 and SRT1720 treatment ($P<10^{-5}$)

[d]

taof(Nrip1) Hypothesis

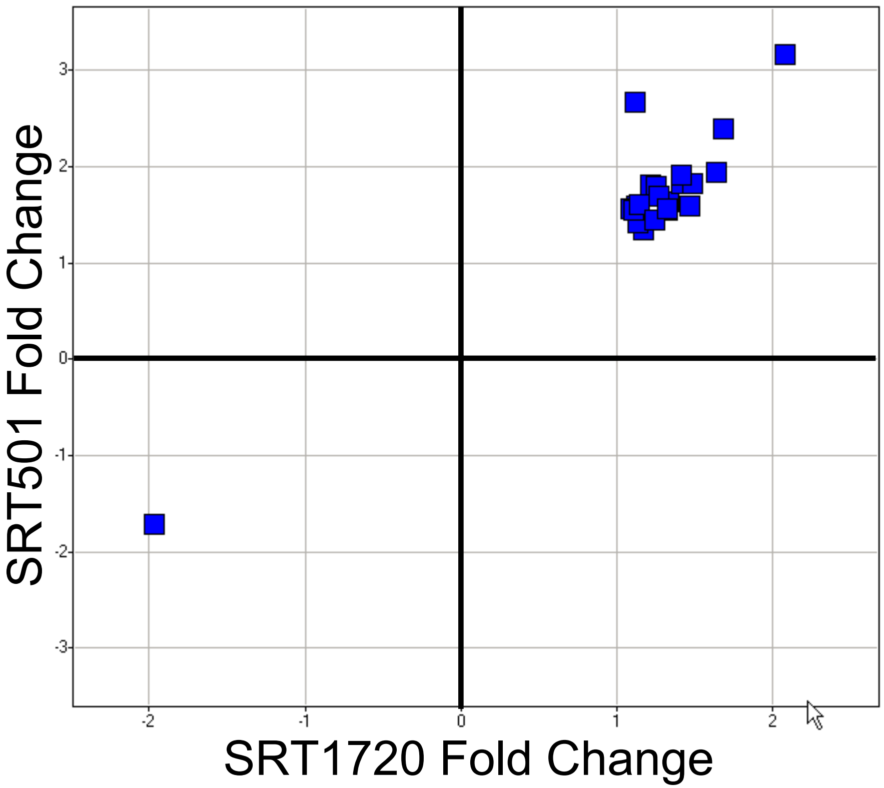

22 of 22 probe sets modulated in same direction after SRT501 and SRT1720 treatment ($P<10^{-5}$)

[e]

taof(Pparα) Hypothesis

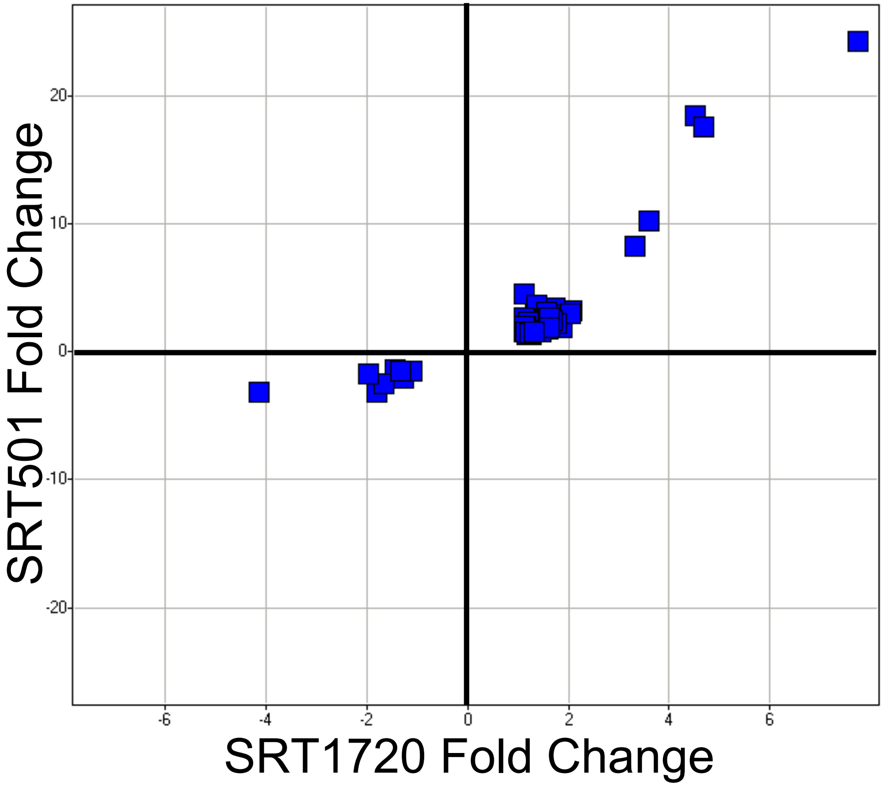

75 of 75 probe sets modulated in same direction after SRT501 and SRT1720 treatment ($P<10^{-5}$)

[f]

taof(Pparγ) Hypothesis

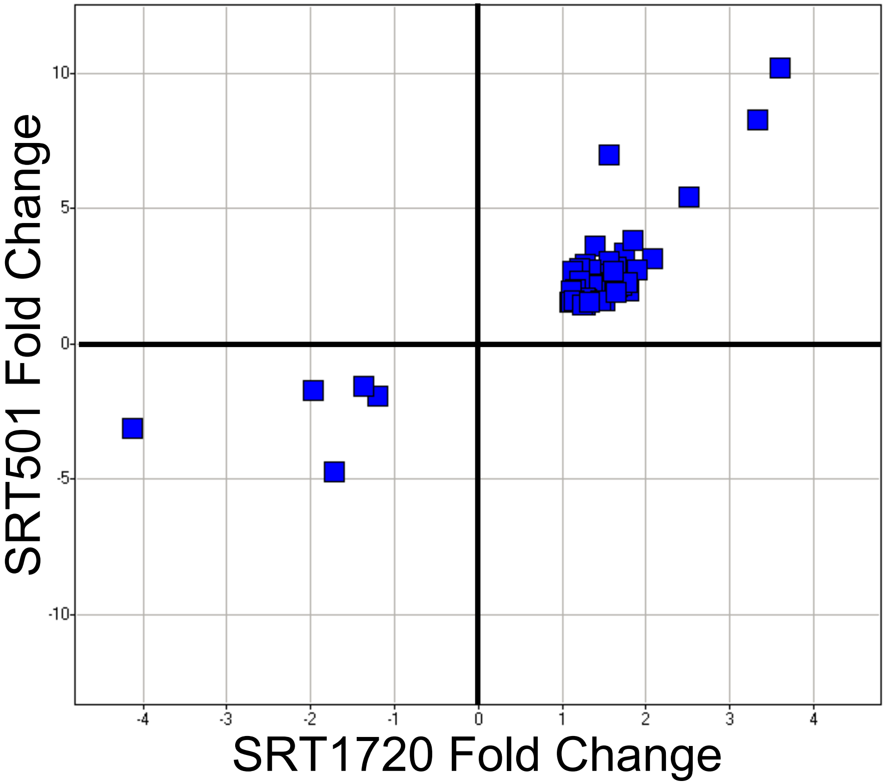

60 of 60 probe sets modulated in same direction after SRT501 and SRT1720 treatment ($P<10^{-5}$)

[g]

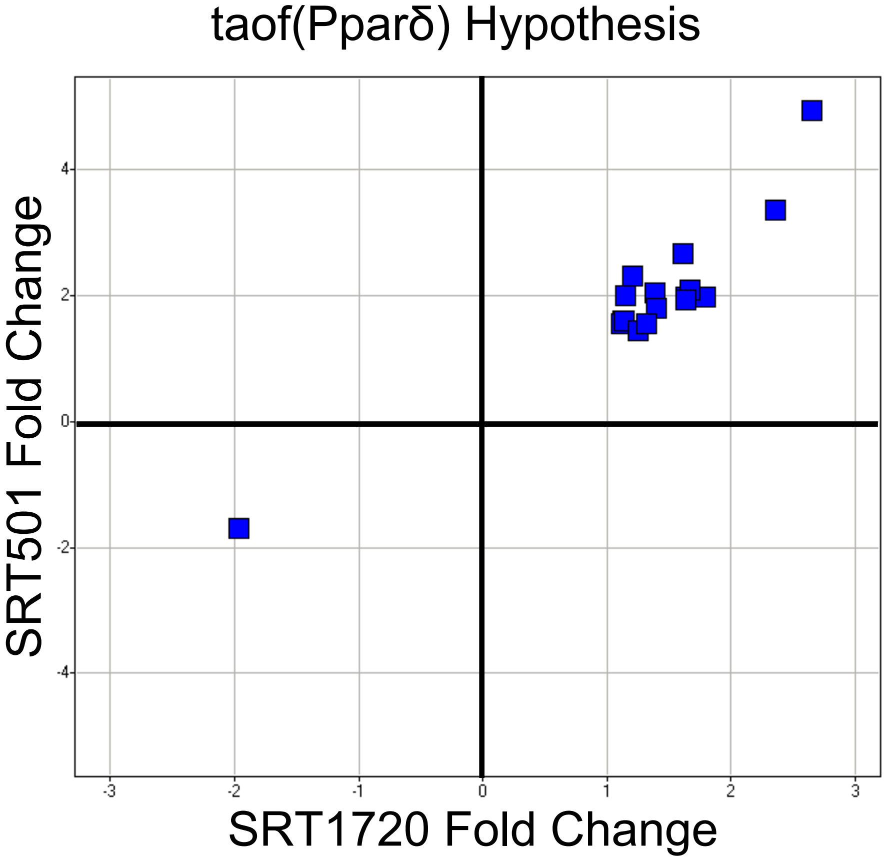

17 of 17 probe sets modulated in same direction after SRT501 and SRT1720 treatment ($P<10^{-5}$)

[h]

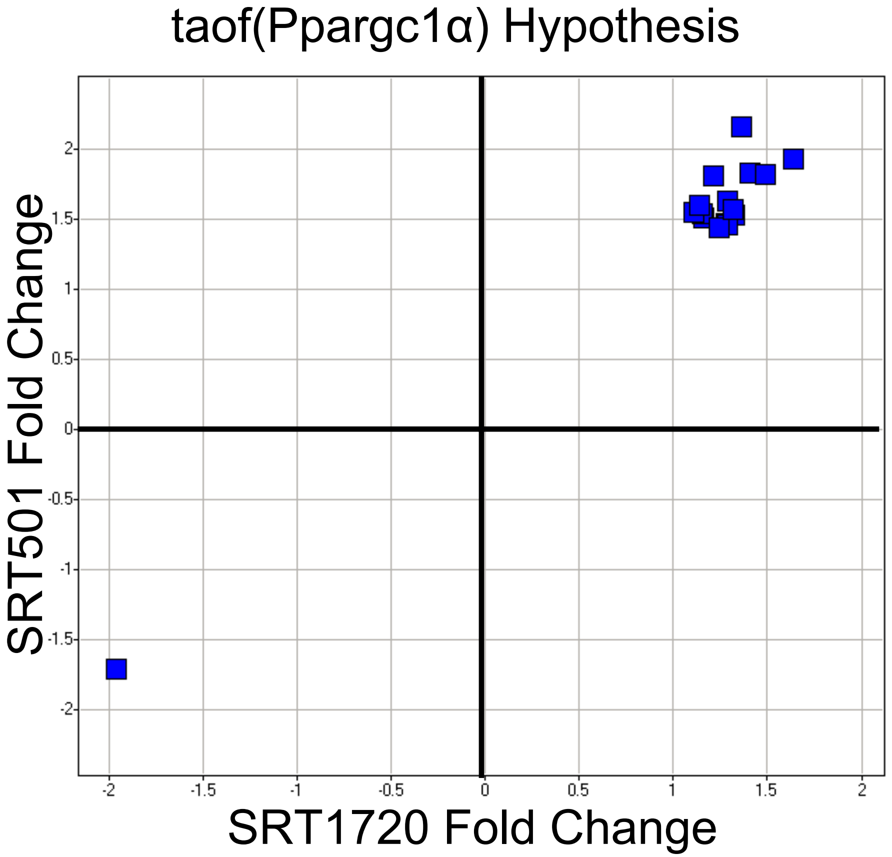

16 of 16 probe sets modulated in same direction after SRT501 and SRT1720 treatment ($P<10^{-5}$)

[i]

Egf Hypothesis

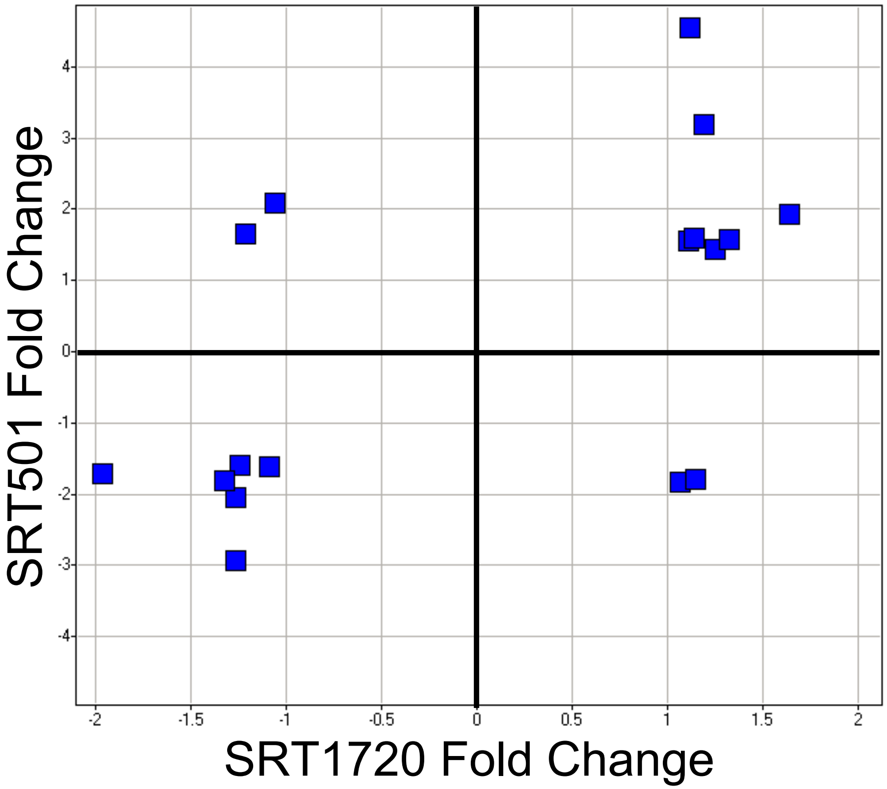

13 of 17 probe sets modulated in same direction after SRT501 and SRT1720 treatment ($P<10^{-5}$)

[j]

taof(NF-κB complex) Hypothesis

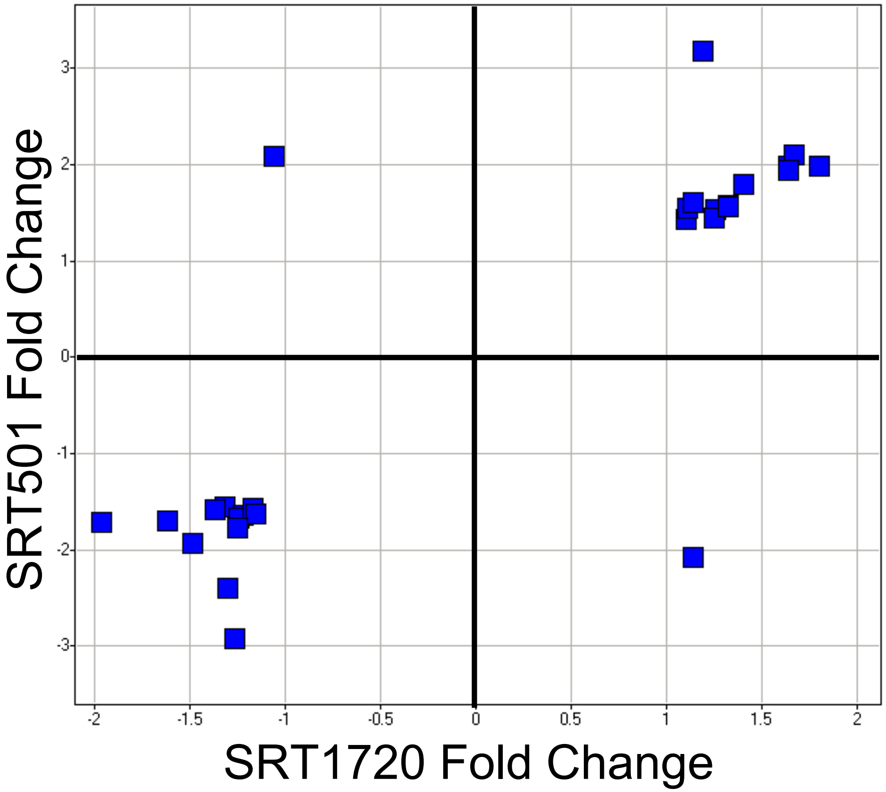

25 of 27 probe sets modulated in same direction after SRT501 and SRT1720 treatment ($P<10^{-5}$)

[k]

taof(Nr3c1) Hypothesis

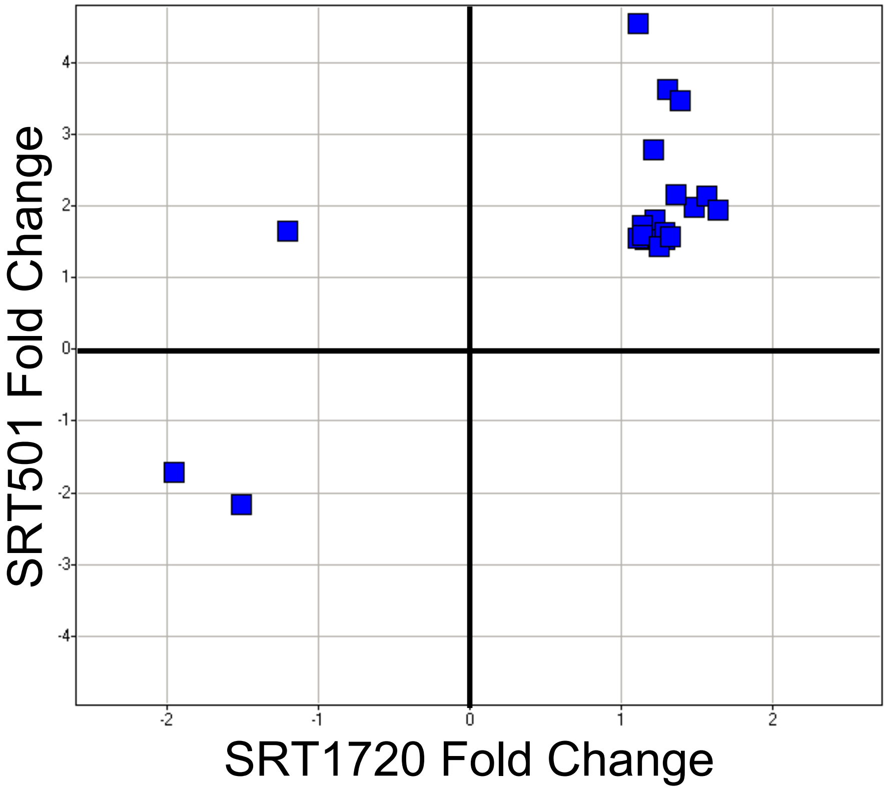

20 of 21 probe sets modulated in same direction after SRT501 and SRT1720 treatment ($P<10^{-5}$)

[l]

Tnfα Hypothesis

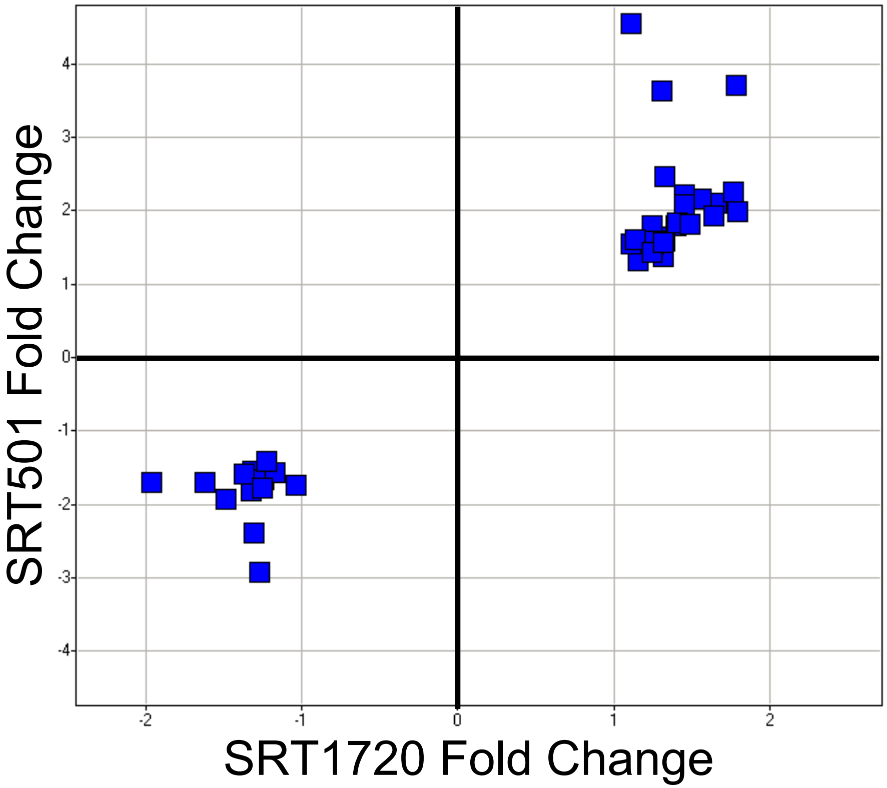

40 of 40 probe sets modulated in same direction after SRT501 and SRT1720 treatment ($P<10^{-5}$)
